# Elucidating protein-protein interactions using the HYDE scoring function

**DOI:** 10.1186/1758-2946-6-S1-P36

**Published:** 2014-03-11

**Authors:** Eva Vennmann, Nadine Schneider, Gudrun Lange, Matthias Rarey

**Affiliations:** 1 Center for Bioinformatics, University of Hamburg, Bundestr.43, 20146 Hamburg, Germany; 2 Bayer CropScience AG, Industriepark Hoechst, G836, 65926 Frankfurt am Main, Germany

## 

Protein-protein interactions take place in every aspect of life. The diversity of those interactions and their significant role in regulatory pathways account for the special interest in them. As shown in various diseases, protein-protein interactions can be deregulated and the cause of illness. Therefore, a better and more detailed understanding of protein-protein interactions is of great importance.

Protein-protein interactions can be classified according to their stability into permanent, long-lasting or transient, functionality-dependent complexes [1]. The latter ones are of special interest for using them to influence regulatory pathways. Deeper insights into protein-protein interactions can be achieved by comprehending the stability of protein-protein complexes and especially of the interface in all its details.

In our study we analyzed the stability of protein-protein interactions using the recently developed HYDE scoring function [2-4]. HYDE consistently describes hydrogen bonds, the hydrophobic effect and polar dehydration and has been proved successful in estimating protein-ligand binding affinities. In this way, HYDE enables to estimate the stability of protein-protein interactions as well as the energetical contribution of single amino acids e.g. to identify so called ‘hotspot’ residues.
